# Examination of the natural mineral water quality in the Kesserwan region, Lebanon

**DOI:** 10.1016/j.heliyon.2024.e33699

**Published:** 2024-06-26

**Authors:** Maud Rizk, Rachelle Atallah, Joya Maria EL Khazen

**Affiliations:** a Department of Nutritional Sciences, Faculty of Health Sciences, University of Balamand, Dekwaneh, Lebanon; b Department of Medical Sciences Laboratories, Faculty of Public Health, Lebanese German University, Sahel Alma, Lebanon

**Keywords:** water quality, contamination, Pollution, Physico-chemical properties

## Abstract

**Introduction:**

Water is an essential element for life, especially the water that we drink. The water is consumable for humans as soon as it meets certain quality requirements. Any chemical, physical, or biological change in water quality may lead to harmful effects on health.

**Objective:**

This study presents the updated situation of some spring water from groundwater in Lebanon, specifically in the Kesserwan region.

**Method:**

To assess the quality of the water, certain physico-chemical parameters such as total dissolved solids, temperature, pH, and microbiological indicators were monitored on 15 sampling sources covering the Kesserwan region. All the parameters were studied during the winter period.

**Results:**

The results identified multiple contaminated sources in Kesserwan. Consequently, groundwater cannot be consumed directly without treatment. Out of the 15 sources tested, only 8 were found to be microbiologically safe, while the remaining 7 were contaminated and required treatment before consumption or use.

**Conclusion:**

Each municipality in the region should be responsible for protecting and maintaining the cleanliness of the areas surrounding the spring water. Additionally, regular, systematic testing of the spring water must be conducted to ensure its suitability for drinking by confirming the absence of contaminants.

## 1 Introduction

Groundwater is one of the most vital natural resources on Earth, playing a crucial role in maintaining ecosystems and supporting human activities. It refers to water located beneath Earth's surface, stored in porous rocks and layers of soil known as aquifers. Groundwater serves as a vital source of fresh water, particularly in regions where surface water is scarce or unreliable [1,2]. In Lebanon, water drawn from wells or springs is used directly for human consumption without any further treatment, and this situation is particularly alarming.

Groundwater pollution occurs when harmful substances, including chemicals, pathogens, and radioactive materials, contaminate the groundwater. This contamination can arise from various sources, such as industrial activities, agriculture, mining, and improper waste disposal. Once pollutants enter the groundwater system, they can persist for years, with far-reaching effects on both human health and the environment [2].

This contamination poses a significant environmental and public health threat, leading to a spectrum of health issues, from cancer to neurological disorders. Furthermore, it can result in substantial economic consequences, including diminished property values and heightened expenses for water treatment and remediation. Preventing groundwater pollution demands a multifaceted approach involving proper waste management, pollution control measures, and effective regulatory frameworks. Additionally, monitoring and testing groundwater quality are essential for identifying and addressing contamination risks. By taking proactive steps to protect our groundwater resources, we can ensure their long-term sustainability and safeguard the health of our communities and the environment [3].

The two major sources of drinkable water are surface water and groundwater. Generally, both forms of water are not safe at their source and require some form of treatment before being considered potable. To ensure adequate water quality, regulatory guidelines exist for biological contaminants (pathogenic bacteria, protozoa, viruses, and helminths), inorganic chemicals (metals, oxyanions, nitrogen species, and radionuclides), and organic chemicals (natural organic matter and synthetic organic chemicals from agricultural, industrial, and residential use). In regions where drinking water undergoes disinfection treatment, both disinfectant residuals and disinfection by-products are regulated to mitigate potential adverse health effects. Additionally, physical aspects of water, including color, odor, and taste, contribute to its quality [4,5].

Water needs are steadily increasing due to factors such as a growing population and modern lifestyles promoting excessive water consumption. Unfortunately, the available water volume is decreasing due to pollution issues, negatively impacting natural water resources. Various types of pollution, including physical, chemical, and biological pollution resulting from the presence of bacteria and viruses, pose significant health risks. Depending on the degree and nature of pollution, polluted waters must undergo different physical, chemical, and biological treatments [6]. In Lebanon, half of the water supply comes from groundwater, primarily from karstic aquifers, which rapidly absorb rain and snow water to form groundwater reserves. Surface waters are abundant and widely distributed throughout Lebanon; nevertheless the residents rely on the groundwater as a crucial source of fresh water [7].

In this article, we will explore the characteristics of groundwater in the Keserwan region, its significance, and the challenges associated with managing this essential resource.

## 2 Materials and methods

### 2.1 Site Description

**Fig. 1:**
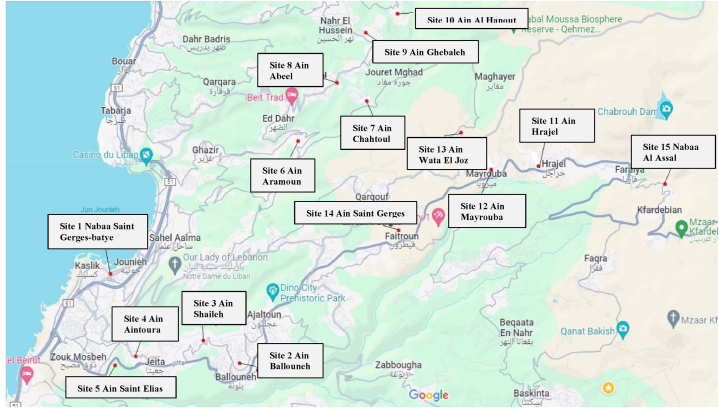
The 15 sampling sites.

This study collected samples in the Keserwan region, in the northeast of Lebanon's capital, Beirut. The 15 locations (Fig. 1) were selected based on altitude, with 5 samples below 650 m, 5 samples between 650 and 1200 m, and 5 samples above 1200 m. Each site was chosen for specific characteristics.

Site 1 “Nabaa Saint Gerges-batye” is located in a public environment in Jounieh at an altitude of 10 m. The municipality of Jounieh takes good care of this place.

Site 2 “Aïn Ballouneh” is located in the center of Ballouneh, far from the habitats, at an altitude of 650 m. The municipality also takes good care of the water and the environment.

Site 3 “Aïn Shaileh” is located in an urban area in Shaileh at an altitude of 550 m, where hygiene rules are respected around this site.

Site 4 “Aïn Aintoura” is located in an overpopulated area in Aintoura at an altitude of 230 m. Wastewater passes near this source, and agricultural land surrounds the area.

Site 5 “Aïn Saint-Elias” is located in a religious and cultivated place in Jeita at an altitude of 380 m.

Site 6 “Aïn Aramoun” is located in a populated area in Aramoun at an altitude of 730 m. In this region, sewage is discharged into open-bottom septic tanks due to the absence of sewage pipes.

Site 7 “Aïn Chahtoul” is located in a populated village and passes near habitats in Chahtoul at an altitude of 920 m. The pollution and domestic waste (cans, plastic bags, and detergent bottles) are remarkably present in this area.

Site 8 “Aïn Abeel” is located in a vacant place in Ghineh at an altitude of 950 m.

Site 9 “Aïn Ghebaleh” is located in a populated area in Ghebaleh at an altitude of 890 m. This region serves as grazing ground.

Site 10 “Aïn Al Hanout” is located near a few houses in Yahchouch at an altitude of 650 m, but the inhabitants respect the rules of hygiene.

Site 11 “Ain Hrajel” is located in a populated area in Hrajel at an altitude of 1320 m. The source is in the center of the village, but the municipality takes good care of the water and the environment.

Site 12 “Aïn Mayrouba” is located in a populated area in Mayrouba at an altitude of 1300 m, where human waste is scattered everywhere.

Site 13 “Aïn Wata El Joz” is located near several farms (chickens, cows, sheep) and cultivated lands in Wata El Joz at an altitude of 1430 m.

Site 14 “Aïn Saint Gerges” is located near the church in a region supervised by the municipality in Feitroun at an altitude of 1200 m.

Site 15 “Nabaa Al Assal” is located in a densely populated area that respects the rules of hygiene in Faraya at an altitude of 1700 m.

### 2.2 Water sampling

During this study, stream samples were taken at 15 points in sterile containers (120 ml and 500 ml) in January 2022. All samples were confidentially collected and preserved according to specified technique and sampling protocol. The date and time of collection were noted on each bottle. Sampling took place in the morning between 8:00 and 10:00 a.m., with careful consideration of sample temperature and seasonal conditions, such as rainfall.

The sampling procedure included washing hands before sample collection, removing the cap without touching the vial's edge or the inside of the cap, securely closing the cap, and avoiding vehicular traffic near the sampling source. Samples were transported to the laboratory within 24 h while maintaining their temperature to perform physicochemical and bacteriological analyses. Each sample was analyzed twice.

### 2.3 Data Description

The studied parameters include three physicochemical parameters (total dissolved solids (TDS), pH, and temperature) and two biological parameters (fecal coliform and total coliform). These parameters were carefully selected based on data availability, significance, and their concentrations in relation to WHO guideline values [5,8].

### 2.4 Laboratory analysis

Laboratory tests were conducted following the “Standard Methods for the Examination of Water and Wastewater” of the American Public Health Association, 1998 Edition.

#### 2.4.1 The color and odor of water sample

The color of each water sample was visually assessed. Unfiltered water from the sample bottle was then agitated to dissolve any soluble substances and then observed under diffused light against a white background. The intensity and tones of colors were recorded. If any insoluble substances were present, they were allowed to settle before examination. The level of color intensity (none, faint, light, or dark) and the shade (e.g., yellow or yellow-brown) were documented [9].

Odor assessment was conducted using the threshold odor test, a conventional method. This involved presenting a series of flasks to an observer, who was informed that some samples might have odors and that the series was organized based on ascending concentrations. Additionally, the observer was provided with a known odor-free blank as a reference during the test. The observer then compared the flasks in increasing order with the blank and recorded if any sample flask emitted an odor [10].

#### 2.4.2 Measurements of pH, Temperature, and TDS

The measurements of pH, temperature, and TDS were conducted on the sampling day. The pH probe was calibrated using pH 7 and 10 buffer solutions. pH was measured using a pH meter model HI 2211, temperature was recorded with a thermometer, and TDS was measured using a TDS meter model HACH 9532700 [11].

#### 2.4.3 Microbiological analyses

Microbiological analyses were conducted to assess various types of bacteria, including total coliforms, *Escherichia coli, Clostridium perfringens,* and total germs. Fecal coliform and total coliform were determined using the membrane filtration technique. Contamination indicator bacteria were detected and enumerated using the membrane filtration method (Büchner method) and by direct culture. The total number of bacteria grown on plate count agar (PCA) was determined at a temperature of 22 °C after incubation for 72 h or at 37 °C for 24 h in 1 ml of water. The Swab test results should not exceed 0-10 adenosine triphosphate (ATP) particles per 100 ml. For bacteria count, the results should indicate the absence of *E. coli,* total or fecal coliforms per 250 ml, and *Clostridium* per 50 ml. Regarding total germs at 22 °C, the count should not exceed 100 colony-forming unit (CFU)/ml, and at 37 °C, it should not exceed 20 CFU/ml [[11], [12], [13], [14]].

## 3 Results and discussion

### 3.1 Organoleptic parameters

The color of the water in all 15 samples was carefully assessed and documented. Changes in water taste and the onset of odors can result from the introduction of various foreign substances, including organic materials, dissolved gases, and inorganic compounds, into the water. These substances can originate from agricultural, natural, and domestic sources, contributing to changes in water quality [10]. Although the samples were not directly tasted, it is crucial to recognize that taste evaluation becomes pertinent only after confirming the water's full potability [11]. It is important to highlight that all of these sources remain in use as potable water by the local inhabitants. Thorough organoleptic water analysis revealed no indications of undesirable color or odor in any of the samples.

### 3.2 Physico-chemical parameters

The water temperature (°C), pH, and TDS (PPM) values from various sites are presented in Table 1.

**Table 1:** Results obtained from the physico-chemical analyses of spring waters.

SOURCES	TDS^a^ (PPM)	pH^b^	Temperature^c^ (°C)
Nabaa Al Assal	176	8.5	8.5
Aïn Wata El Joz	308	8.20	9
Aïn Saint Gerges	108	8.26	10
Aïn Hrajel	378	7.43	9
Aïn Shaileh	282	8.01	13
Aïn Ballouneh	283	7.91	13
Aïn Mayrouba	480	8.20	10
Aïn Abeel	241	7.32	13
Aïn Ghebaleh	380	7.61	12
Aïn Chahtoul	298	7.42	14
Aïn Aramoun	435	7.61	12
Aïn Al Hanout	331	7.64	14
Aïn Aintoura	424	7.47	15
Aïn Saint-Elias	428	8.05	14
Nabaa Saint Gerges-batye	298	7.85	15

a TDS levels should be ≤ 500 PPM to ensure palatability and prevent excessive scaling.

b pH levels of municipal water should be between 6.5 and 8.5 to be safely consumed.

c Normal water temperature should range between 10 °C and 15 °C.

According to standard, pure water should have a pH of 7 [5], but source waters tend to be slightly acidic due to precipitation and water pollutants. The pH measurements in the spring waters remained relatively stable, ranging between 7.32 and 8.5. This slight increase in pH with a basic tendency could be attributed to the dissolution of carbonate from limestone rocks with water [7,15].

The levels of dissolved solids in spring water remained relatively stable. However, certain spring waters, such as “Ain Mayrouba,” " Ain Aramoun,” " Ain Saint-Elias, " and " Ain Aintoura” exhibited slightly higher TDS values, indicating an external supply of mineral salts. These salts may originate from the ground following human activities, such as domestic water discharges, or from areas where the rocks contain a high concentration of salt.

According to international standards, the temperature at all sites should be between 10 °C and 15 °C [11]. However, three sources, namely “Ain Wata El Joz”, “Ain Hrajel”, and “Nabaa Al Assal,” exhibited some disturbances with temperature below 10 °C. These disturbances may occur during rainy weather conditions. Subsequently, the water temperature decreases due to altitude and reduced exposure to sunlight. These findings support previous research by Hamid Bou Saab and colleagues, who found that water temperature at sampling sites along the “Nahr Ibrahim” river in the Kesserwan region varies with altitude and season [16]. Similar variations were noted in a study conducted in the lower Litani basin during summer, where temperatures exceeded 15 °C, particularly given the hot climate of the Bekaa region [7].

### 3.3 Microbiological analysis

Total coliforms represent a category of bacteria that naturally occurring on plants, in soils, and in the intestines of humans and warm-blooded animals. While the majority of these bacteria are not of fecal origin, their detection in treated water does not necessarily indicate an immediate threat to public health. However, their presence can serve as an indicator of water quality deterioration and the potential presence of human enteric viruses. *E. coli,* a member of the coliform group, stands out as the sole bacterium exclusively found in the intestines of mammals, including humans, and certain animals [17].

Bacteriological analyses showed several sources, including “Nabaa Al Assal,” "Aïn Saint Gerges,” "Aïn Hrajel,” "Aïn Saint-Gerges,” "Aïn Ballouneh,” "Aïn Al Hanout,” "Aïn Shaileh,” and "Nabaa Saint Gerges-batye,” had natural mineral waters that are microbiologically safe due to the absence of the studied bacteria. The protection of the surrounding region, maintenance, and cleanliness of these areas, make these sources safe for consumption and culinary use.

**Fig. 2:**
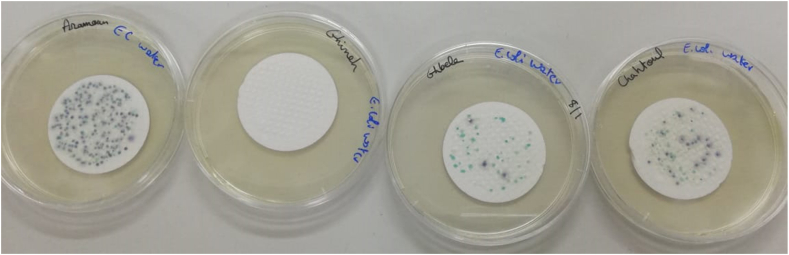
Colonies of *E.coli* and Coliforms cultured after incubation for 24 h at a temperature of 37 °C in 250 ml of "Aïn Aramoun, " "Aïn Abeel, " "Aïn Ghebaleh, " and "Aïn Chahtoul " springs.

**Fig. 3:**
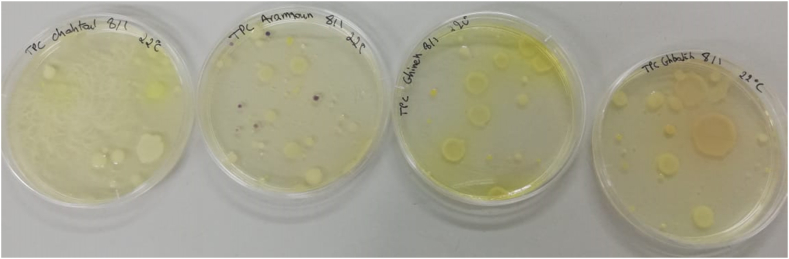
Total numbers of germs cultured on PCA medium after incubation for 72 h at a temperature of 22 °C in 1 ml of "Aïn Aramoun, " "Aïn Abeel, " "Aïn Ghebaleh, " and " Aïn Chahtoul” sources.

**Fig. 4:**
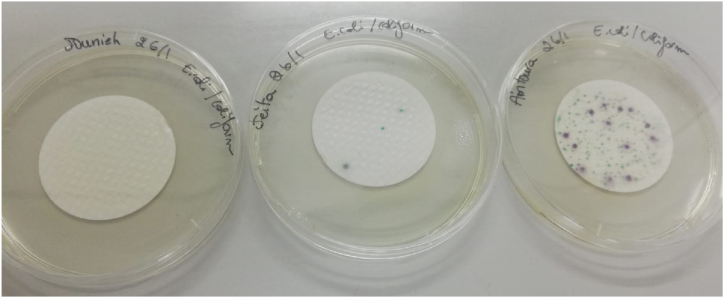
E. coli and Coliform colonies grown on Rapid E. coli II medium after membrane filtration and incubation at 37 °C for 24 h in the "Nabaa Saint Gerges-batye,” "Aïn Saint-Elias,” and "Ain Aintoura” sources.

Following the enumeration of these bacteria, seven sites were found to be infected with total coliforms. These same sites, with the exception of "Ain Mayrouba”, were also found to be contaminated with *E. coli and Clostridium perfringens* (Table 2 and Fig. 2, Fig. 3, Fig. 4).

**Table 2:** Results obtained from the bacteriological analyses of spring waters.

Source	Swab Test	*E. coli*	*Total/fecal coliforms*	Total Germs		*Clostridium*
				22 °C	37 °C	
Nabaa Al Asal	4	0	0	0	0	0
Aïn Wata El Joz	29	1	3	34	2	4
Aïn Saint Gerges	0	0	0	0	0	0
Aïn Hrajel	0	0	0	0	0	0
Aïn Shaileh	0	0	0	0	0	0
Aïn Ballouneh	0	0	0	0	0	0
Aïn Mayrouba	25	0	11	55	9	0
Aïn Abeel	8	0	0	60	0	0
Aïn Ghebaleh	0	3	57	100	7	1
Aïn Chahtoul	14	22	56	132	2	1
Aïn Aramoun	119	96	42	205	33	4
Aïn Al Hanout	0	0	0	0	0	0
Aïn Aintoura	36	13	122	380	90	1
Aïn Saint-Elias	31	1	6	24	1	1
Nabaa Saint Gerges-batye	0	0	0	0	0	0

Some sites, such as "Aïn Mayrouba,” had high levels of Coliforms due to the presence of wastewater passing near the source. Elevated germ values on the PCA medium indicate the presence of other unstudied bacteria or unidentified organisms in the water. Bacteriological contamination was also observed at "Ain Wata el Joz.”, likely due to its proximity to the cultivated land using fertilizers and nearby farms. Similarly, the "Aïn Saint-Elias” site showed microbiological contamination, possibly related to the proximity of cultivated fields using fertilizers. The "Aïn Ghebaleh” site showed high water contamination levels, indicated by the elevated number of *E. coli,* coliforms and *Clostridium* bacteria. This suggests long-standing fecal contamination due to poor hygiene practices near the source, which serves as pastureland. At "Aïn Chahtoul” and "Aïn Aintoura” sites, bacterial dynamics were increasingly changing, with significant fecal contamination observed at "Aïn Chahtoul” due to domestic discharges in this area. The two sources, "Ain Aintoura” and "Ain Aramoun,” were identified as polluted and undrinkable, presenting a high health risk due to waterborne diseases. The major causes of contamination include discharge from nearby residential units and the lack of treatment plans. The municipality plays a major role in the protection and cleanliness of the area surrounding the spring water, as shown in the meta-analysis conducted by Genter et al. on the importance of government presence in protecting source waters from fecal contamination [18].

On the other hand, the "Nabaa Al Asal” source was microbiologically safe but presented some ATP molecules, possibly linked to handling errors. The source "Ain Ghebaleh,” despite containing bacteria, showed a negative Swab test, likely attributed to water chlorination by the municipality. The Swab tests showed high values in sources with microbial contamination, especially in "Ain Aramoun.” These spring waters must be treated to protect the inhabitants of the region from poisoning.

Several criteria contribute to this contamination: firstly, the high number of inhabitants in the region, which increases the rate of pollution and human waste; Secondly, the lack of maintenance and facilities to manage wastewater pipes; and finally, the lack of protection and cleanliness around some springs, with garbage and animal waste, increasing the risk. Similar results were identified in the North region, where the quality of groundwater was unacceptable and contaminated by sewage due to the absence of municipal maintenance and cleaning of surrounding areas [19].

Finally, several limitations faced our study. The first essential limitation of our study is the impossibility of measuring all pathogenic microorganisms in spring water due to several factors, including the cost of microbiological tests and the shortage of equipment. The second limitation is the inability to test all the spring waters present in the Kesserwan region, given their abundance.

## 4 Conclusion

The existing literature on spring waters in the Kesserwan lacks comprehensive studies and tests. Therefore, this study is significant as it conducted an analysis of specific characteristics and the microbiological quality of the primary spring waters in the area.

This study provides valuable information on natural mineral waters and spring waters in Kesserwan, offering citizens insights into water quality and facilitating better choices. This work is based on physico-chemical parameters and microbiological analyses to ensure the potability of spring water in the region. The results of this work identify several contaminated sources, indicating that groundwater cannot be directly consumed without treatment. Consequently, each region must take responsibility for systematically testing spring water to ensure the absence of contaminants. Additionally, the area surrounding the spring water must remain protected and clean from human and animal waste. Finally, the municipality has an important role in ensuring healthy and well-maintained wastewater facilities.

This study highlights the importance of further research to explore and identify additional physico-chemical parameters and other bacterial strains present in these waters, utilizing more efficient methods.

This research did not receive any specific grants from funding agencies in the public, commercial, or not-for-profit sectors.

## Data availability statement

Data was included in article/supp. Material/referenced in article.

## CRediT authorship contribution statement

**Maud Rizk:** Writing – review & editing, Writing – original draft, Validation, Supervision, Methodology. **Rachelle Atallah:** Methodology, Formal analysis. **Joya Maria EL Khazen:** Resources, Investigation.

## Declaration of competing interest

The authors declare that they have no known competing financial interests or personal relationships that could have appeared to influence the work reported in this paper.
